# The Potential Elimination of *Plasmodium vivax* Malaria by Relapse Treatment: Insights from a Transmission Model and Surveillance Data from NW India

**DOI:** 10.1371/journal.pntd.0001979

**Published:** 2013-01-10

**Authors:** Manojit Roy, Menno J. Bouma, Edward L. Ionides, Ramesh C. Dhiman, Mercedes Pascual

**Affiliations:** 1 Department of Ecology and Evolutionary Biology, University of Michigan, Ann Arbor, Michigan, United States of America; 2 Howard Hughes Medical Institute, University of Michigan, Ann Arbor, Michigan, United States of America; 3 Faculty of Public Health and Policy, London School of Hygiene and Tropical Medicine, University of London, London, United Kingdom; 4 Department of Statistics, University of Michigan, Ann Arbor, Michigan, United States of America; 5 Fogarty International Center, National Institutes of Health, Bethesda, Maryland, United States of America; 6 National Institute of Malaria Research, Delhi, India; University of California San Diego School of Medicine, United States of America

## Abstract

**Background:**

With over a hundred million annual infections and rising morbidity and mortality, *Plasmodium vivax* malaria remains largely a neglected disease. In particular, the dependence of this malaria species on relapses and the potential significance of the dormant stage as a therapeutic target, are poorly understood.

**Methodology/Principal Findings:**

To quantify relapse parameters and assess the population-wide consequences of anti-relapse treatment, we formulated a transmission model for *P. vivax* suitable for parameter inference with a recently developed statistical method based on routine surveillance data. A low-endemic region in NW India, whose strong seasonality demarcates the transmission season, provides an opportunity to apply this modeling approach. Our model gives maximum likelihood estimates of 7.1 months for the mean latency and 31% for the relapse rate, in close agreement with regression estimates and clinical evaluation studies in the area. With a baseline of prevailing treatment practices, the model predicts that an effective anti-relapse treatment of 65% of those infected would result in elimination within a decade, and that periodic mass treatment would dramatically reduce the burden of the disease in a few years.

**Conclusion/Significance:**

The striking dependence of *P. vivax* on relapses for survival reinforces the urgency to develop more effective anti-relapse treatments to replace Primaquine (PQ), the only available drug for the last fifty years. Our methods can provide alternative and simple means to estimate latency times and relapse frequency using routine epidemiological data, and to evaluate the population-wide impact of relapse treatment in areas similar to our study area.

## Author Summary

*Plasmodium vivax*, one of the four species responsible for malaria in humans, represents today a much larger global public health problem than previously recognized. The neglect of *P. vivax* is still apparent in the limited efforts made so far to understand the regional dynamics of the disease, and in the non-existence of practical models to guide and evaluate control measures. One main impediment is the overall uncertainty in the dynamic role of the hidden reservoir, resulting from the liver stage known as hypnozoites and responsible for relapses, a characteristic feature of this species. Current control efforts and elimination plans are therefore hampered in their ability to quantify the impact of effective anti-relapse treatments. This paper applies a *P. vivax* transmission model to surveillance data from a semi-arid region (in NW India), whose clear demarcation of the transmission and relapse seasons makes it especially suited for estimating relapse parameters such as frequency and rate. The model results quantify the striking dependence of *P. vivax* on relapses for its survival, supporting the feasibility of regional elimination and lending support to the expressed urgency of replacing Primaquine, the anti- relapse drug now in use for over fifty years with unsatisfactory efficacy, resistance and side-effects.

## Introduction

Recent years have seen an increasing appreciation of the significance of *Plasmodium vivax* as a threat to global health. Estimates of the population at risk of the disease have been upgraded to between 2.5 and 2.6 billion, and the number of annual infections to 130–435 million [1]. The parasite has re-emerged in areas previously cleared of the disease [2], and reports of serious morbidity and mortality have proved this form of malaria to be less benign than previously assumed [3], [4]. Resistance to Chloroquine (CQ) appears to be spreading, and serious concerns exist about the efficacy of Primaquine (PQ), the only drug in use for relapses for over 50 years [5]. With the persisting gaps in our understanding of its transmission dynamics (despite rare theoretical studies [6], [7]) and mode of action of available treatments, *P. vivax* malaria is among the world's most neglected diseases [2]–[4], [8]. In particular, central questions remain on the role of relapses in its population dynamics, especially on the importance of treating hypnozoites to interrupt transmission [9].

Of the human malaria parasites, only *P. vivax* and *P. ovale* have the ability to delay the development of a fraction of the infectious load of sporozoites in the liver. The activation of dormant hypnozoites results in the relapse of the disease after the primary infection is cleared from the bloodstream. Although mechanisms of activation are still unknown [10], they appear to serve the parasite in conditions less favorable for transmission, such as the short season of suitable temperatures and available vectors in high latitudes [11]. The need for an improved treatment that would target these dormant stages has been raised [9], with the reported spread of both tolerance and resistance to PQ [12]–[14]. Furthermore, studies suggest low patient compliance for the full 14–day PQ course, particularly among poor patients [15] and the shortened 5-day course, designed to limit side-effects in G6PD (Glucose-6-Phosphate Dehydrogenase)-deficient patients, is of limited or no use [12], [13], [15], [16]. Besides these deficiencies in current treatment, the size of *P. vivax'*s hidden reservoir poses a serious challenge to eliminating the disease, with relapses known to occur for up to 5 years [2], [17].

In particular, difficulties in evaluating the effectiveness of anti-relapse treatment [5], [9], [18] have contributed to both the continued use of PQ despite weakening effectiveness, and our inability to quantify the potential public health benefits of more effective alternatives. Operational difficulties arise in following treated patients for prolonged periods of time, with the possibility of confounding re-infections [10]. The study of relapse patterns is further complicated in areas of high transmission by strains with short latency times and higher relapse rates, and the likely accumulation and re-activation of hypnozoites from many previous infections [19], [20].

Low transmission regions provide an opportunity to assess the latency time and the fraction of relapsing infections, and to quantify in a dynamical way the population level consequences of relapses and of their treatment. In these regions, *P. vivax* strains typically exhibit fewer relapses and longer latency times [14], and relapses and transmission tend to dominate different seasons [21]. This is the case in the Indian subcontinent, in the arid and semi-arid areas of northwest India, where *P. vivax* typically exhibits two seasonal peaks of which the first is dominated by relapses, as we will further substantiate below, and the second is shared with *P. falciparum* and reflects mainly transmission following the monsoon rains [21].

## Methods

### Data

The malaria data come from Kutch, a large arid district in the state of Gujarat, located at the desert fringes of northwest India with seasonal epidemic malaria [22], [23]. The data were provided by the Commissionerate Health of the Government of Gujarat, through the National Institute of Malaria Research in India, and consist of monthly confirmed *P. vivax* cases from blood slides of patients visiting public health services between January 1987 and August 2010. Because of marginal environmental conditions for transmission determined by the semi-arid climate in the region, interannual variation in rainfall generates strong yearly variation in epidemic intensity, as we have shown earlier for the transmission of *P. falciparum* [23], [24]. Monthly rainfall data were recorded at the local weather station in Kutch, and supplied by the Indian Meteorological Department in Pune (India). Yearly population data were obtained via interpolation of the decadal census data between 1991 and 2011.

### Transmission model

**Figure 1:**
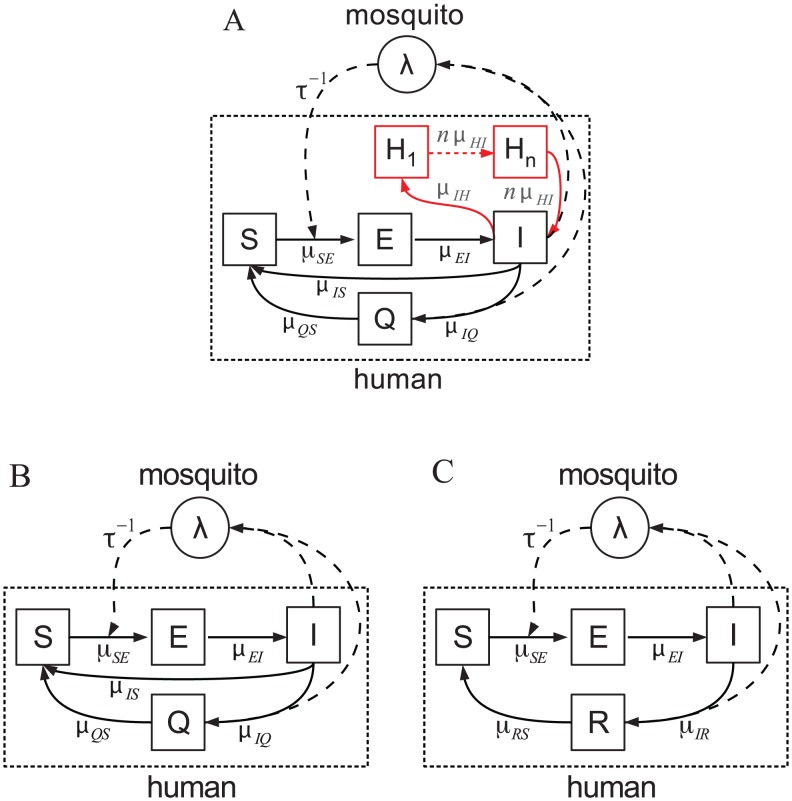
*P. vivax* transmission models. Human classes are represented as squares, and mosquito class *λ* as a circle, with arrows indicating the direction of transition between classes. The per-capita rate of transition is included next to each arrow (see supplement for model equations and description). A. The general model SEIH*^n^*QS with *n* H classes and the relapsing loop I-to-H_1…*n*_-to-I (in red). The multiple H classes effectively implement a realistic Gamma-distributed latency period [25], and may also represent possible intermediate stages of hypnozoites during its long stay in the liver (see supplement for more details). The class Q provides a simple representation of partial, temporary, immunity that confers protection against clinical infection but not infectiousness [40]. Prompt clinical intervention prevents some patients from developing immunity [41], which is captured in the I-to-S transition. B. The non-relapse model SEIQS, without the relapse loop but otherwise identical to SEIHnQS. C. The non-relapse model SEIRS, where the completely immune class R (no blood-stage parasitemia) replaces the Q class, and the I-to-S transition is no longer present. (This model is same as the VSEIRS model previously used for the population dynamics of *P. falciparum* malaria in Kutch [23]).

The human component of our *P. vivax* transmission model is formulated by dividing the population into classes based on infection status (Fig. 1A): S, for naïve individuals who are susceptible to both infection and disease; E, for exposed or inoculated humans who are not yet infectious; I, for infectious humans that can transmit the pathogen to the vector; Q, for individuals that have acquired some degree of immunity from disease and are therefore more weakly infectious than those in class I; and H_1…*n*_, a chain of *n* dormant classes for humans who carry liver-stage hypnozoites. In view of the existing lack of knowledge about the metabolism of hypnozoites, the chain of H classes allow us to both represent putative stages of the hypnozoite in the liver and generate a more realistic distribution of the duration of the overall liver stage [25] (see Supplement for details). We consider three H classes, and hereafter refer to this particular model as SEIH^3^QS (Fig. 1A). Models with fewer H classes fit the data poorly, and those with more classes do not have enough statistical support to justify the added complexity (see below).

**Figure 2:**
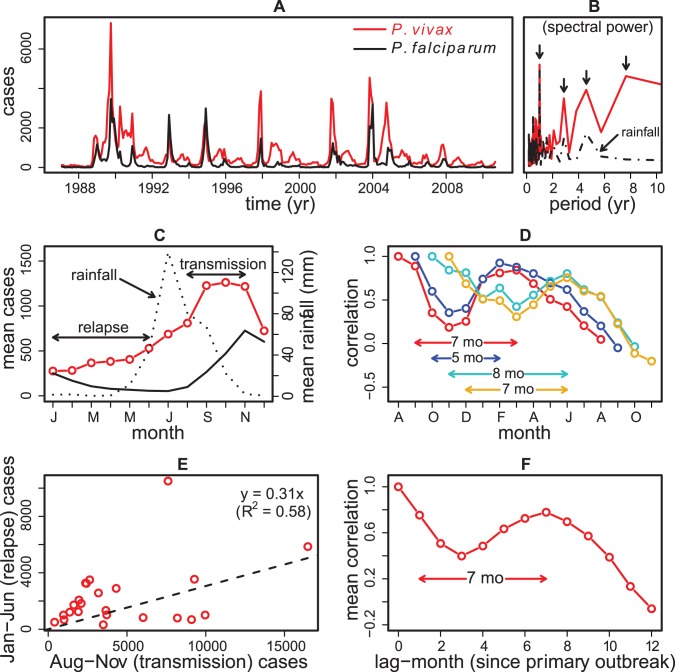
Malaria time series and regression estimates of relapse frequency and latency. A. The monthly *P. vivax* (red) and *P. falciparum* (black) cases between January 1987 and August 2010 are overlaid. B. The power spectrum of *P. vivax* data (red) shows a strong yearly cycle as well as different dominant periods longer than one year (i.e. interannual cycles) (The power spectrum describes how the variance of the data is distributed among different periods). The interannual cycles of period less than 6 years are also present in the rainfall spectrum (dashed black). C. The comparison of the observed seasonal patterns of *P. vivax* (red) and *P. falciparum* (solid black) shows significant relapses in *P. vivax* during January–June when rainfall is minimal (dotted black) – these are called “relapse cases” in the text. The majority of *P. vivax* cases due to transmission arise between August and November, and are likewise referred to as “transmission cases”. D. The autocorrelation values of *P. vivax* cases in each of the four transmission months - August (red), September (blue), October (light blue) and November (yellow) - and the cases in the subsequent twelve months show large correlation peaks between January and June, giving latency periods ranging between 5 and 8 months. E. A linear regression of aggregated transmission and relapse cases gives a slope of 0.31, suggesting a 31% average relapse rate. In the driest months of May–June, when entomological conditions exclude transmission, the regression gives a much tighter relation (not shown). F. The average of these autocorrelation curves suggests a relapse latency of 7 months.

In the absence of mosquito data and also keeping our inference goal in mind, the vector dynamics is incorporated implicitly in terms of its force of infection, *λ*, and a Gamma-distributed lag time with mean *τ* to account for the developmental delay of *P. vivax* parasites within surviving mosquitoes (see supplement). Variability in the dynamics arises from fluctuations in mosquito abundance and behavior, and is incorporated with three exogenous forces: seasonality (to account for yearly periodic forces), rainfall (as a climate covariate), and environmental noise (see supplement, and also [23]). Seasonality of transmission is represented by a set of flexible functions (see supplement for details) and therefore, is not specified *a priori*. The shape and timing of this component of transmission is freely determined from the data in the process of fitting the model. Although below we refer to the “relapse” and “transmission” seasons, based on the seasonal pattern of monthly cases relative to that of the monsoon rains (Figure 2), this distinction is only used in correlative analyses and in comparisons between data and model outputs.

To incorporate anti-relapse treatment in the SEIH^3^QS model, we assume that a fraction *a* of the patients (in the I class) are effectively treated of relapses and move to the Q class, and the untreated fraction 1 – *a* enters the dormancy stage via the I-to-H_1_ transition (as in the control model; see Eq.S1 in supplement). The fraction *a* reflects the percentage of new *P. vivax* infections for which relapses are fully suppressed; we use this variable to express different levels of relapse treatment below. The transition of all treated humans to the Q class, rather than directly to the S class, follows from our assumption that the hypothetical anti-relapse drug is effective only against hypnozoites and does not affect any residual blood-stage parasitemia. This is a reasonable assumption given the activity of a typical hypnozoitecide [12], [13], [19].

### Model simulation

The model with maximum-likelihood-estimated parameters (see below) is simulated several years ahead, and for many realizations of surrogate rainfall time series (generated from the original rainfall data by extending the standard amplitude-adjusted, fast-fourier-transform based method, so as to preserve the seasonality pattern in Fig. 2C (dotted curve), besides the autocorrelation and power spectrum). To quantify the effect of treatment on *P. vivax* transmission, we compute the percent reduction in transmission cases between Aug-Nov relative to control. To compute the elimination probability of *P. vivax* malaria after *T* years, we simulate the treatment model for an extensive period of time (up to 1000 yrs ahead, *T*<1000) and compute the probability that not a single *P. vivax* case arises between *T* and 1000 yrs. We note that treatment does not represent here the administration of primaquine, but of a hypothetical, not-yet-existing, more effective, anti-relapse compound. Ideally, such a drug would be an improvement on the long treatment regime Primaquine requires, and formulated in a way it can be administered in conjunction with a drug directed against blood stages (e.g. CQ).

### Statistical inference

We formally relate the model to data by assuming that only a fraction *ρ* of new infections are detected by the surveillance methods [23], and model the data as negative binomially distributed around these new infections (see Supplement for details). (We note that only the transitions from E-to-I and H_3_-to-I contribute to the observed cases, and that individuals in Q are considered completely asymptomatic. This does not mean that all individuals in I are necessarily symptomatic as variation within that class in symptom levels can partly explain the under-reporting.)

To estimate model parameters and test different models given the data, we carried out likelihood-based inference via a recently introduced iterated filtering methodology [26] for calculating maximum likelihood estimates (MLEs). Applications of this methodology to other epidemiological models can be found in the literature [23], [24], [27]–[29]. To investigate the potential weak identifiability of some parameters (resulting from including more parameters than the data can support), we computed confidence intervals for each MLE by using the profile likelihood method [23], [24], [28]. We used the R package “pomp” [30] to implement the algorithm, which is detailed elsewhere [23], [27], [28].

The SEIH^3^QS model is tested against two other relapse models with one and six H classes, denoted by SEIHQS and SEIH^6^QS respectively (Fig. 1A). A single H class generates exponentially distributed latency periods, whereas multiple classes have the advantage of allowing for a flexible (gamma) distribution [25]. To ascertain the importance of relapses in explaining the data, the SEIH^3^QS model is also compared with two non-relapse models, SEIQS and SEIRS (Fig. 1B–C). These comparisons are carried out in terms of the Akaike Information Criterion (AIC), a likelihood-based selection criterion that penalizes higher model complexity.

## Results

### Patterns in the data

*P. vivax* cases show epidemic patterns characterized by strong yearly and interannual fluctuations (Fig. 2A), with periodicities shorter than 6 years mirroring those of rainfall (Fig. 2B). Most malaria cases for both parasite species typically occur between August and November following the annual July-to-September monsoon, and the lowest number of cases occur during the colder pre-monsoon months (Fig. 2C). Conditions for transmission before the monsoon are poor, as shown in the decline of reported cases for *P. falciparum* (Fig. 2C). The high autocorrelation between reported *P. vivax* cases in the pre-monsoon season and those in between August and December (Fig. 2D) indicates that the pre-monsoon season mainly reflects relapses, with estimated latency period between 5 and 8 months. In particular, during the 2 driest months (May and June) the variance of cases explained by cases in October and November is 85% (not shown). Thus, reported cases from January to June can be used to estimate the fraction of cases between August and November that relapse. A linear regression of transmission and relapse cases gives a relapse rate of 31% (Fig. 2E), suggesting that, on average, one in three *P. vivax* patients (who recovered from the primary infection) have clinical relapses in the following spring. This estimate is close to the 28% relapse rate observed in the neighboring Kheda district [14], [31] and the national average of 30% [32]. Cases show significant autocorrelation peaks between the months of August-November and those from January to June, providing an estimated latency period between 5 and 8 months (Fig. 2E), and a mean value of 7 months (Fig. 2F). This relatively long interval is compatible with the “temperate” strains of *P. vivax,* rather than the “tropical types” with short and frequent relapses found in more endemic parts of India [14], [33].

### Model comparison and parameter estimation

The comparison of the models without relapses (SEIQS and SEIRS) to those with relapses (SEIHQS, SEIH^3^QS and SEIH^6^QS), shows that the data do not support the non-relapsing models (Table 1). The SEIH^3^QS model is clearly superior to both non-relapse models. Moreover, the models with multiple H classes perform better than the one with a single H class. For parsimony, the simplicity of the model SEIH^3^QS is preferred over the model SEIH^6^QS with six H classes.

**Table 1:** A likelihood-based comparison of the fitted models.

Model	log-likelihood *L*	*P*	AIC	likelihood ratio test
SEIH^3^QS (relapsing, with three H classes)	−1809	26	**3670**	–
SEIH^6^QS (relapsing, with six H classes)	−1805	29	**3668**	–
SEIHQS (relapsing, with one H class)	−1815	24	3678	–
SEIQS (non-relapsing)	−1824	21	3690	*p*≪0.001
SEIRS (non-relapsing)	−1830	19	3698	*p*≪0.001

Log-likelihood (*L*) and number of estimated parameters (*P*) are given for the different models. The model selection criterion known as AIC (for Akaike Information Criterion) is computed as AIC = −2*L*+2*P*. A likelihood ratio test comparing each of the two non-relapse models SEIQS and SEIRS to the best relapse model (SEIH^3^QS) rejects these models (based on a chi-square test with 5 and 7 degrees of freedom respectively). The models with multiple dormant classes perform better than the one with a single class. For parsimony, the model SEIH^3^QS with three such classes is preferred over the model SEIH^6^QS with 6 classes (see Methods, Supplementary Material, for a description of the models).

**Figure 3:**
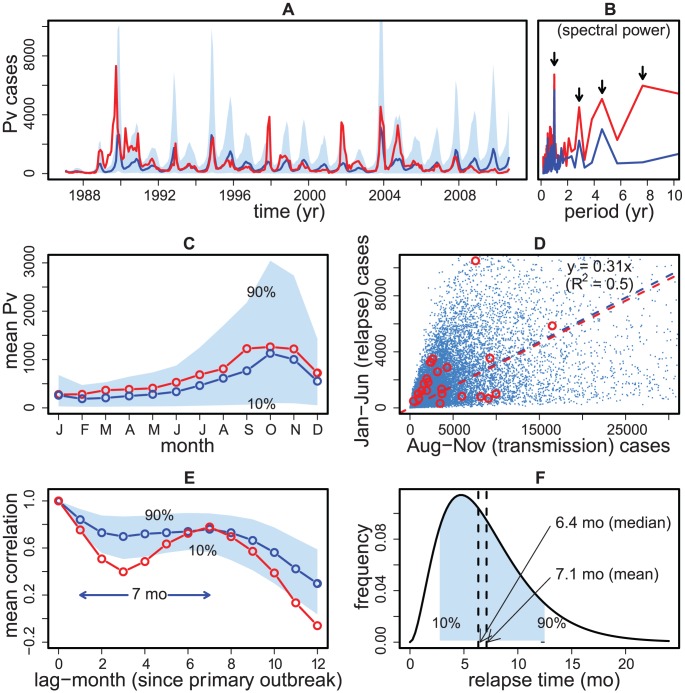
Comparison of model simulations with malaria data and regression estimates. The data and the regression estimates for relapse latency and frequency from figure 2 are plotted in red, the median of 1000 model simulations is plotted in blue, and the 10–90 percentile range shaded in light blue (all simulations use the maximum-likelihood-estimates of the parameters, see suppl Table S1). A. The peaks and troughs of the monthly simulated cases track the data well, except for the underestimation and overestimation of large outbreaks before and after 2007 respectively (see text). B. The power spectrum of the median cases from the simulated time series reproduces the yearly and the two longer interannual cycles observed in the data (and also present in the rainfall covariate, see Fig. 2B). C. The seasonal patterns in the data and simulations show good agreement. D. A linear regression of aggregated transmission and relapse cases in each of the 1000 simulation time series gives the same 31% relapse rate as in the data. E. The average autocorrelation curve of the simulated cases reproduces the observed 7-month mean latency period. F. The gamma-shaped latency distribution is plotted using the shape parameter values taken from supplement Table S1, which predicts median and mean latency periods of 6.4 and 7.1 months respectively.

Figure 3 illustrates the performance of the best model (SEIH^3^QS) relative to the observed seasonal pattern in the malaria data. The observed and simulated time series exhibit peaks and troughs that fall on the same months during most years (Fig. 3A), and have similar dominant periods of their inter-annual cycles (Fig. 3B). Such agreement is encouraging given that these simulations are not one time-step ahead predictions, but they rely instead only on estimated initial conditions for January 1987 to compute the entire 23-yr long trajectories. The model tends to underestimate the large outbreaks prior to 2007 (which explains the overall lower mean in Fig. 3C), and to overestimate them afterward. This reversal may reflect a shift in the vector control policy adopted in 2007 [34], which was not included in the model. Simulations also reproduce the observed 31% relapse rate and 7-mo mean latency period (Figs. 3D,E). In particular, the mean latency is well predicted by the model's distribution for the latency period whose respective median and mean are 6.4 and 7.1 months (Fig. 3F).

In contrast, the tested non-relapse models (SEIQS and SEIRS) widely miss the two patterns observed in the data (supplement, Fig. S2), consistent with their poor statistical fit (Table 1). Thus relapses clearly play a significant role in the dynamics of *P. vivax* malaria in this region. This role becomes more evident when the effect of rainfall is taken into consideration: pre-monsoon (relapse) and post-monsoon (transmission) cases reveal a remarkably strong correlation ( = 0.90) during years of low monsoon rain (supplement, Fig. S3), indicating that relapses are more vital for the parasite survival after drier, low, transmission years.

### Effects of anti-relapse treatment

**Figure 4:**
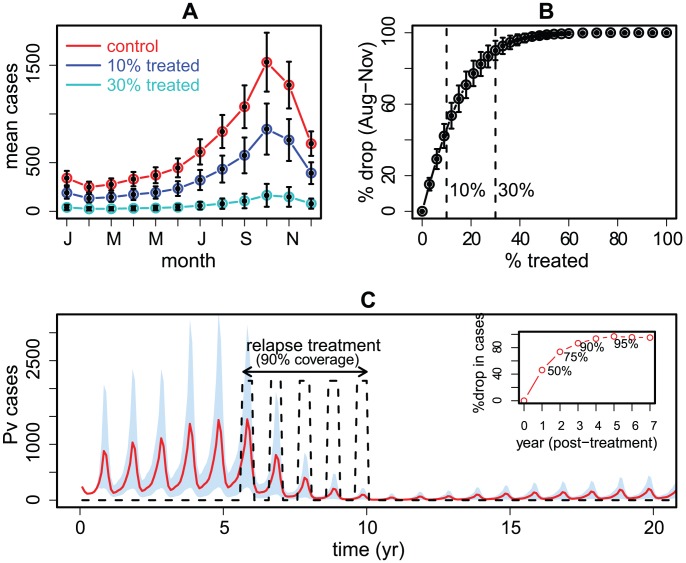
Model simulations with an effective anti-relapse treatment. For a given treated fraction *a*, the model is driven by a surrogate rainfall series (reproducing main features of the observed rainfall data) and simulated 25 yrs ahead, with 1000 independent runs per surrogate and 1000 surrogate series in all (see Methods, Supplementary Material). The error bars denote standard errors over surrogates. A. The median seasonal patterns are compared among control (red), 10% treatment (blue) and 30% treatment (light blue), showing a drop of 45% and 90% in transmission cases (aggregated over Aug–Nov) relative to control for these two treatment levels. B. Treatment effect, defined as the relative drop in transmission cases, as a function of *a*. C. In the alternative annual mass treatment scenario, with 90% of the population successfully covered at the beginning of the year for 5 consecutive years from year 6-to-10 (equivalent to *a* = 0.9 between January of year 6 and December of year 10, and *a* = 0 at other times), the *P. vivax* burden is substantially reduced on average, ranging from a 50% drop after one annual intervention, to 95% at the end of the treatment period (the median of the surrogate runs is plotted in red, and the 10–90 percentile range shaded in light blue; the inset shows the cumulative % reduction in the *P. vivax* burden after mass treatment). Without further prevention of relapses the parasite will gradually recover over several decades, highlighting the need to use such an intervention in conjunction with effective treatment of clinical cases.

The striking dependence of *P. vivax* on relapses can provide a much-needed analytical tool for assessing the population-wide benefits of clinically targeting hypnozoites [5]. Figure 4 summarizes key findings from simulating the SEIH^3^QS model with a hypothetical, improved [9], [35], relapse treatment (see Methods), taking current clinical practices in the study area as a baseline. When implemented for 25 years, the prevention of 10% of relapses alone can suppress transmission in the post monsoon period by as much as 45%, and a 30% treatment doubles this effect (Fig. 4A–B). The gradual weakening of outbreak sizes in successive years further suggests the possibility of completely eliminating the parasite, with the effective treatment of 65% of relapses resulting in the extinction of *P. vivax* within a decade (Figs. S4). As these delivery rates could be beyond a country's operational capacity, particularly in more endemic areas, we consider the impact of an annual mass treatment with a coverage rate of 90% implemented just after the transmission season in January (Methods). We conservatively estimate that such an intervention, would on average reduce the *P. vivax* burden considerably (Fig. 4C). Moreover, an effective relapse treatment level can have a bigger impact when transmission in the previous year was low (e.g. cumulative cases below 1000 between August and November), with the prevention of 10% relapses reducing cases in the following transmission season by as much as 70% (supplement, Fig. S4).

## Discussion

We formulated a dynamic model of *P. vivax* transmission, and applied novel statistical methods for parameter inference from routine surveillance data, to characterize the relapsing patterns in a low-endemic region in NW India. Our findings reveal a clear dependence of the parasites on relapses for survival in an area with seasonal transmission. They also quantify population-wide impacts, including potential elimination of the parasite, of an effective anti-relapse treatment. The striking dependence of *P. vivax* on relapses demonstrated with our model supports the hypothesis [5], [9] that this stage represents the Achilles heel of this malaria species, and emphasizes the urgent need to develop more effective relapse treatments than the currently available Primaquine [9], [13], [18].

Our model identifies a single relapse within one year, in agreement with clinical studies in the region [14], [31], [33]. Reported longer latency times, as long as 5 years [10], should not have a major impact on our estimated elimination times as treatment that targets all hypnozoites will also prevent most relapses that would have stayed dormant for any period in excess of one year. Although it is not feasible to quantify and remove the effects of an existing 5-day PQ regime [14] (only recently replaced by a full 14 day course [36]), any resulting bias is expected to be small, given the inadequacy of the 5-day course [15], [16] and the small fraction of total cases currently treated with PQ [37].

The observation that the impact of a relapse treatment is more marked when the cases from the preceding transmission season are low, has clear operational implications. It suggests a more cost-effective strategy that would rely on the intermittent application of treatment only in low-transmission years, where resources are limited and the development of drug resistance is a concern. This result further implies that combining an anti-relapse treatment with a more effective blood schizonticide (than CQ) would have a larger impact than the sum of each individually, as treating new infections would limit sexual reproduction and subsequent infections during the same season. Note that this interaction is in addition to the augmented curative effect of PQ when administered with a schizonticide (CQ) on a patient level [5].

More generally, the above observation implies that the effective treatment of hypnozoites will have most impact when used in conjunction with other intervention measures that reduce transmission, such as effective vector control. As both rainfall and vector control influence transmission, the combined use of effective vector control with an effective relapse treatment should act synergistically. Because the fitted model implicitly includes current levels of vector control in the transmission rate *β* (see Supplement) any explicit implementation of interventions that reduce this value in the model should be interpreted as additional to the baseline of current practices. To illustrate the synergy of these two modes of intervention, we implemented a reduction in the fitted transmission rate of the form *β*_new_ = (1−*c*) *β* during the monsoon months, when the mosquito population tends to rise and current IRS efforts are concentrated. Small increases of current control levels can significantly decrease the treatment coverage needed for elimination in a given time horizon. For example, the 65% coverage needed, under existing vector control practices, for the total (100%) suppression of *P. vivax* transmission and its elimination in a decade (supplement, Fig. S4-B), can be brought down to 30% with *c* = 0.1 (that is, with *β*_new_ = *β*×0.9).

The eradication attempts in the 1960s combined the attack on vector and the parasite. However, maintaining gains and eliminating the silent parasite reservoir in the population proved a difficult challenge. Effectively removing relapses could play an important role in overcoming these limitations encountered in the past. The synergy we inferred in this study between hypnozoite removal and transmission reduction further highlights the desirability of a judicious mix of control methods to address new initiatives to eliminate malaria.

Our approach has relied on the pronounced variability of *P. vivax* malaria in a region of unstable transmission where rainfall acts as a strong environmental determinant at seasonal and interannual time scales [23]. Seasonal transmission of malaria limited by rainfall or temperature is a dominant feature of the global epidemiology of malaria. Relapses provide malaria species such as *P. vivax* with the ability to bridge periods unfavorable for transmission, and potentially, with a competitive advantage when coexisting with non-relapsing species. Our results in desert fringes of NW India should generalize to the extensive temperate regions in South America and Asia where *P. vivax* dominates. In areas with more intense, seasonally extended or perennial transmission, the higher relapse frequency and shorter latency times of prevalent *P. vivax* strains could signify an adaptation to competition between strains of *P. vivax* or with *P. falciparum*. If relapses provide a competitive advantage in areas of extended transmission, blocking relapses could also have beneficial control implications. However, these effects cannot be easily extrapolated from our results, and require investigation with time series from these regions. Although the model formulation is sufficiently flexible to consider different relapsing characteristics, such as shorter latency times and multiple relapses, the ability of our approach to infer relapse parameters from surveillance data will obviously decrease with weaker seasonality. Thus, future work should investigate how far beyond highly seasonal, low endemic regions this approach can be fruitfully applied.

Our estimates of the impact of treatment depend on the ability of the fitted model to separate relapses from primary infections. This ability should be reflected in confidence intervals of the estimated parameters and this uncertainty is taken into consideration when simulating the model to evaluate the effect of treatment. Independent information on the seasonality of transmission could be used to further pin down the dynamics of relapses by specifying this seasonality rather than completely inferring it from the data. Obtaining such information from the transmission dynamics of *P. falciparum* in the same region would appear plausible, but is not straightforward, as the two species have different temperature requirements, benefiting *P. vivax* in the cooler seasons.

The simplification adopted by considering a single partially immune (Q) class in our model ignores the details of an age–stratified immunity distribution, but still proves sufficient in capturing the transmission dynamics in this epidemic region. Our results provide little statistical support for the Q class itself, given the large confidence intervals of related parameter estimates, which effectively renders this class unnecessary to explain the observed data. In more endemic areas, where host immunity from repeated infection is likely to play a more important role [10], the immune (Q) class may find support from the data.

Although our model makes no assumption about the symptomatic or asymptomatic nature of relapses, it does consider that all relapses contribute to infections that can potentially be counted as cases (*i.e.* infections in class I). To allow for relapses that do not contribute to cases, as well as for completely asymptomatic relapses, we have considered a variant of the model in which only a fraction *f* of relapses transition into I, and the remaining (1−*f*), into Q. The relapse parameters and findings on the effects of treatment proved robust to this variation.

Finally, our analysis illustrates that retrospective surveillance data, in combination with simple models of malaria transmission (e.g. [38]) and recent statistical developments for confronting these models with data, can complement operationally complex clinical studies of relapses. In particular, as shown here, the variable incidence patterns in time series of *P. vivax* malaria from seasonal transmission regions provide valuable information on the dynamical role of relapses and the potential effect of anti-relapse treatments. Simple mechanistic models thus offer a foundation for more detailed formulations as additional data become available [39].

## Supporting Information

Text S1 **Model description.** Details of model description, including equations, are included in the supplement.
(PDF)

Figure S1 **Correlation between cases and accumulated rain.** The correlation is significantly higher in the months between June and October (solid line + circle) during the monsoon season, than when all twelve months are considered (dashed line + triangle), due to the confounding effects of relapse cases in the latter.
(PDF)

Figure S2 **Comparison between data (red) and models SEIH^3^QS (blue), SEIQS (yellow) and SEIRS (green).** Each model is simulated 1000 times. A. A linear regression of aggregated transmission and relapse cases gives relapse rates of 34% and 35% for the two non-relapse models SEIQS and SEIRS, respectively, both values higher than the 31% rate estimated from the SEIH^3^QS model and suggested by the data. Data points (red open circles) are also shown. B. The SEIQS model has a shorter mean latency of 5 months, (and the SEIRS model by construction has none), compared to the 7-mo latency estimated by the SEIH^3^QS model and consistent with the data.
(PDF)

Figure S3 **Correlation of relapse and transmission cases.** The SEIH^3^QS model is simulated (using the MLE parameters) 25 yrs ahead with 1000 surrogate rainfall series, and Jan–Jun relapse cases, and the Aug–Nov transmission cases are aggregated for each simulated year, giving 25,000 paired values. A. A scatterplot of these (aggregated) relapse and transmission case pairs does not exhibit a meaningful correlation because of the confounding effects of rainfall. B. Partitioning by low (red) and high (blue) rainfall years, defined by total monsoon rain (aggregated between June–Sept) < and >75 mm, reveals a strong positive correlation during low-rainfall years. C. The (aggregated) rainfall distribution has a median of 75 mm, giving the same number of red and blue points in figure B.
(PDF)

Figure S4 **Relapse treatment and elimination probability.** Elimination is computed as the year-long absence of *P. vivax* malaria cases for every year starting in a given year and lasting for the entire simulation length ( = 1000 yrs). A. Representative examples of simulated Pv time series for the three treatment levels used in Fig. 3A, *a* = 0, 0.1, 0.3, using a single surrogate rainfall. Progressive suppression of cases at 30% treatment level (light blue) suggests the possibility of *P. vivax* elimination in near future. B. Plot shows mean year of *P. vivax* elimination as a function of treatment level (*a*), indicating that 40% treatment can eliminate the parasite in 25 years, and 65% treatment can achieve the same within a decade (mean and error bars are computed over 200 surrogate rainfall series). C. Plot shows the probability of elimination in 25 yrs (circles) and 10 yrs (stars), versus treatment level, corroborating that 40% and 65% relapse treatment can eliminate *P. vivax* malaria in 25 yrs and 10 yrs respectively (at probability *P* = 0.5).
(PDF)

Figure S5 **Correlation of treatment effect and preceding transmission intensity.** The treatment model S1 is simulated (using MLE parameters) at a 10% treatment level (*a* = 0.1), 25 yrs ahead with 1000 surrogate rainfall series. A. A scatterplot of % drop in current transmission intensity vs preceding (aggregated) transmission cases shows a negative correlation. This inverse relationship arises from a strong positive correlation between these earlier transmission cases and current relapse cases (B), which in turn correlate negatively with the % drop (C), presumably because of the parasite's dependence on relapse to survive during the low-transmission season.
(PDF)

Table S1 **Parameter definition and maximum likelihood estimates (MLEs) for the different models, SEIH^3^QS, SEIH^6^QS and SEIQS.** Parameters not estimated are denoted with superscript ^*^. Setting *μ*_EI_ = 24/yr assumes an average 15-day human incubation period before clinical symptoms arise [11].
(PDF)

Table S2 MLEs for the SEIH^3^QS parameters and their lower and upper bounds of approximate 95% confidence intervals (see Table S1 for units; pre-set parameters are denoted with superscript ^*^. CI values of 0, 1 and ∞ correspond to confidence intervals reaching the boundaries of parameter space.
(PDF)
