# Supplementary material for: The Potential Elimination of Plasmodium vivax Malaria by Relapse Treatment: Insights from a Transmission Model and Surveillance Data from NW India
Source: PLoS Negl Trop Dis. 2013 Jan 10;7(1):e1979. doi: 10.1371/journal.pntd.0001979 (PMC3542148; doi:10.1371/journal.pntd.0001979)
Supplement: Text S1 — Model description. Details of model description, including equations, are included in the supplement. (PDF) [file pntd.0001979.s001.pdf]

Supplementary Text

Similarities in the basic transmission pathways involving the human host and the mosquito vector in *P. falciparum* and *P. vivax* malaria motivated us to build the vivax transmission model upon the recently developed falciparum model for the same region [1, 2], by adding a relapsing component [3]. Our goal is to formulate a population–level model that can capture key aspects of human, parasite and vector dynamics, while remaining parsimonious enough for biologically relevant parameters to be estimated directly from available surveillance data on monthly cases over time. Our modeling framework differs from the classic Ross–MacDonald formulation [4, 5, 6] that assumes constant human and mosquito populations, because in epidemic regions mosquito abundances and malaria cases vary significantly. Therefore we model numbers, and not fractions, of infections.

The basic SEIH^{n}QS model (without relapse treatment) is a special case of the full model that includes treatment, and so we present below equations for the latter (see Table S1 for parameter definitions):

$$\begin{aligned}
\mathrm{d}S/\mathrm{d}t &= (\delta P + \mathrm{d}P/\mathrm{d}t) + \mu_{IS} I + \mu_{QS} Q + (a\mu_{IH} I + b\mu_{EI} E) - \mu_{SE} S - \delta S,\\
\mathrm{d}E/\mathrm{d}t &= \mu_{SE}(t) S - \mu_{EI} E - \delta E,\\
\mathrm{d}I/\mathrm{d}t &= (1-b)\mu_{EI} E + n\mu_{HI} H_n - (\mu_{IH} + \mu_{IS} + \mu_{IQ}) I - \delta I,\\
\mathrm{d}H_1/\mathrm{d}t &= (1-a)\mu_{IH} I - n\mu_{HI} H_1 - \delta H_1,\\
\mathrm{d}H_i/\mathrm{d}t &= n\mu_{HI} H_{i-1} - n\mu_{HI} H_i - \delta H_i \quad [\text{for} \quad i = 2, \cdots, n],\\
\mathrm{d}Q/\mathrm{d}t &= \mu_{IQ} I - \mu_{QS} Q - \delta Q\,.
\end{aligned} \tag{S1}$$

The birth rate for the S class is set to ensure that $S(t)+E(t)+I(t)+Q(t)+\sum_k H_k(t) = P(t)$, where $P(t)$ is Kutch population size at time t obtained from interpolated census data. The advantage of using multiple $\mathrm{H}_i (i = 1, \cdots n)$ classes, which partitions the single dormant stage into a sequence of identical stages (Fig.1A), is to introduce a flexible Gamma–distribution for the relapse intervals s (Fig.3F) given by the formula $G(s) = (n\mu_{HI})^n s^{n-1} \exp\left(-n\mu_{HI}\right)/\Gamma(n)$ [7], where $n\mu_{HI}$ denotes the transition rate from one H stage to the next.

Relapse treatment reduces, by a fraction a, the rate μ_{IH} at which the infected population enters dormancy via I–to–H_1 transition, and the treated humans join non–relapsing infecteds

in moving to the Q class. Equations for the control (untreated) SEIH^{3}QS model, with $n = 3$, thus readily follow by setting $a = 0$.

The role of mosquitoes in the transmission from infected to susceptible humans is represented implicitly through their effect in generating a distributed time delay between the current rate of transmission experienced by a susceptible human at time t, $\mu_{SE}(t)$, and the force of infection resulting from levels of infection in the human population at all previous times [1] . Because this force of infection, λ, determines the number of infected vectors, but does not affect transmission until the parasite completes its development (sporogony) within the surviving mosquitoes over this time, we refer to λ as the "latent" force of infection. Thus, the inoculation rate $\mu_{SE}(t)$ can be expressed as the integral over time of this force of infection, weighted by a probability that describes the delay resulting from parasite development, as follows:

$$\mu_{SE}(t) = \int_{-\infty}^{t} \gamma(t-s)\lambda(s)\mathrm{d}s\,, \qquad \text{with } \gamma(s) = \frac{(k/\tau)^k s^{k-1}}{(k-1)!}\exp(-ks/\tau)\,. \tag{S2}$$

For the delay probability function $\gamma(s)$ we choose a gamma distribution (with mean τ and variance τ^2/k), because it allows us to implement a flexible shape, in particular one with a characteristic time scale of parasite development [7], in contrast to an exponential distribution.

Moreover, the latent force of infection is given by

$$\begin{aligned} \lambda(t) \; &= \; \beta(t)\left[\frac{I + q\,Q}{P}\right], \quad \text{with } \beta(t) = \bar{\beta} \times \text{seasonality} \times \text{rainfall} \times \text{noise} \qquad \text{(S3)} \\ & \qquad = \bar{\beta} \times \exp\left[\sum_{i=1}^{m} b_i s_i(t) + b_r C(t)\right] \times \left[\frac{\mathrm{d}\Gamma(t)}{\mathrm{d}t}\right], \end{aligned}$$

where q denotes a reduced infection risk from humans in the Q class, and the transmission term $\beta(t)$ includes three extrinsic drivers corresponding, respectively, to (1) seasonality, (2) rainfall (as a climatic covariate, see Methods), and (3) environmental noise (encompassing stochastic variability not included in the rainfall), all of which are assumed to affect the vector dynamics. To represent the yearly periodic pattern in a flexible way, seasonality is implemented by $m(= 6)$ periodic spline functions $s_i(t)$. Stochasticity is incorporated by a

Gamma distribution $\Gamma(t)$ with intensity σ^2_{pro} (see [1, 2] for the details of implementing s_i and Γ). We construct the rainfall function $C(t)$ by accumulating rain over the preceding 4 months for each reported month, and then setting January–May and November–December rain to zero. Thus, $C(t) = \int_{t-4/12}^{t} r(s)ds$ between July and October and $= 0$ for other times, where $r(s)$ is a spline interpolation of the discrete (monthly) rainfall data. This particular choice follows from observed correlation properties between monthly vivax cases and rainfall (Fig.S1). We then normalize $C(t)$ to zero mean and unit variance before using it in (S3).

For numerical convenience we do not implement the integral in (S2), and instead use the equivalent representation in terms of sequential transitions through a chain of identical stages, whose only purpose is to generate the desired distributed delay between λ and μ_{SE} [7]:

$\kappa_1, \cdots, \kappa_k (\equiv \mu_{SE})$ as follows [7]:

$$
\begin{aligned}
\mathrm{d}\kappa_1/\mathrm{d}t &= (\lambda - \kappa_1)k\tau^{-1}, \qquad\qquad \text{(S4)}\\
\mathrm{d}\kappa_j/\mathrm{d}t &= (\kappa_{j-1} - \kappa_j)k\tau^{-1}, \qquad \text{for} \quad j = 2, \cdots, k-1\\
\mathrm{d}\mu_{SE}/\mathrm{d}t &= (\kappa_{k-1} - \mu_{SE})k\tau^{-1}.
\end{aligned}
$$

We use $k = 2$ as in [1, 2], which reduces (S4) to equations for κ_1 and μ_{SE}.

Although humans belonging to the E, I and Q classes can all carry liver–stage hypnozoites, we only consider the I–to–H–to–I transition to denote relapse (Fig.1A). This assumption was based on the following considerations: 1) clinical studies designed to determine important relapse patterns, such as relapse rate and frequency, typically track patients through relapsing episodes [8, 9, 10, 11, 12, 13], and can be directly modeled with an I–to–H–to–I type loop; 2) model parsimony dictated by our inference goal limits the amount of unobserved details that time–series data can support [14]; and, 3) if relapse is primarily triggered by physiological stress from illness, as recently suggested [13], the E–to–H and Q–to–H transitions will mostly give non–relapsing humans who have no effect on transmission, and can therefore be safely ignored in the model. Note that this relapse loop includes the possibility of multiple relapses (albeit at a constant rate) as is common in India [9, 15].

The equation for the measurement model, which couples the continuous–time dynamics

of model (S1) with the discrete–time sequence $y_1, \cdots, y_N$ of monthly reported case data at times $t_1, \cdots, t_N$, is given by,

$$y_k \sim \text{NegBin}(M_k, \sigma^2_{\text{obs}}) \quad \text{for} \quad M_k = \rho \int_{t_{k-1}}^{t_k} [\mu_{EI} E(s) + n\mu_{HI} H_n(s)]\, ds\,. \tag{S5}$$

"Negbin(a, b)" is the negative binomial distribution with mean a and variance $a+a^2b$ (see [1] for more details). We carried out all numerical simulations in the R computing environment [16], and used the R package "Pomp" [17] to implement the algorithm for statistical inference, which is detailed elsewhere (e.g. see Supplement of [1] for a summary of the algorithms steps, and also [18]).

Equations of the SEIHQS model, with a single H class, can be obtained by removing the $\text{H}_{k=2\cdots n}$ equations from the untreated version of (S1) and substituting $n = 1$ in (S1) and (S5). The equations for the two non-relapse models can be obtained from (S1) as follows: model SEIQS by setting $\mu_{IH} = \mu_{HI} = 0$, and SEIRS by setting $q = \mu_{IH} = \mu_{HI} = \mu_{IS} = 0$, Q≡R.

Supplementary References

[1] Laneri K, Bhadra A, Ionides EL, Bouma M, Dhiman RC, Yadav RS and Pascual M (2010), Forcing versus feedback: epidemic malaria and monsoon rains in northwest India. *PLoS Comp Biol* 6(9), e1000898. doi:10.1371/journal.pcbi.1000898.

[2] Bhadra A, Ionides EL, Laneri K, Pascual M, Bouma M and Dhiman RC (2011), Malaria in northwest India: data analysis via partially observed stochastic differential equation models driven by Lévy noise. *J Am Stat Assoc* 106, 440–451. doi:10.1198/jasa.2011.ap10323.

[3] Pongsumpun P and Tang I–M (2007), Mathematical model for the transmission of Plasmodium vivax malaria. *Intl J Math Mod Meth App Sci* 1, 117–121.

[4] Ross R (1915), Some a priori pathometric equations. *Br Med J* 1, 446–447.

[5] MacDonald G (1957), The epidemiology and control of malaria. London, Oxford Univ Press.

[6] Smith D and McKenzie FE (2004), Statics and dynamics of malaria infection in Anopheles mosquitoes. *Malaria J* 3, 13.

[7] Lloyd AL (2001), Destabilization of epidemic models with the inclusion of realistic distributions of infectious periods. *Proc R Soc Lond* B 268, 985–993.

[8] Srivastava HC, Sharma SK, Bhatt RM and Sharma VP (1996), Studies on *Plasmodium vivax* relapse pattern in Kheda District, Gujarat. *Indian J Malariol* 33, 173–179.

[9] Adak T, Sharma VP and Orlov VS (1998), Studies on the *Plasmodium vivax* relapse pattern in Delhi, India. *Am J Trop Med Hyg* 59, 175–179.

[10] Huldén L, Huldén L and Heliövaara K (2008), Natural relapses in *vivax* malaria induced by *Anopheles* mosquitoes. *Malaria J* 7, 64.

[11] Gupta S, Gunter JT, Novak RJ, Regens JL (2009), Patterns of *Plasmodium vivax* and *Plasmodium falciparum* malaria underscore the importance of data collection from private health care facilities in India. *Malaria J* 8, 227.

[12] Hanf M, Stéphani A, Basurko C, Nacher M and Carme B (2009), Determination of the *Plasmodium vivax* relapse pattern in Camopi, French Guiana. *Malaria J* 8, 278.

[13] White N (2011), Determinants of relapse periodicity in *Plasmodium vivax* malaria. *Malaria J* 10, 297.

[14] Bretó C, He D, Ionides EL and King AA (2009), Time series analysis via mechanistic models. *Ann App Stat* 3, 319–348.

[15] Joshi H, Prajapati SK, Verma A, Kangá S and Carlton JM (2008), *Plasmodium vivax* in India. *Trends Parasitol* 24, 228–235.

[16] R Dev Core Team (2011), R: a language and environment for statistical computing. *R Foundation Stat Computing*, URL http://www.R-project.org.

[17] King AA, Ionides EL, Bretó C, Ellner S and Kendall B (2009), Pomp: statistical inference for partially observed Markov processes. URL http://pomp.r-forge.r-project.org.

[18] King AA, Ionides EL, Pascual M and Bouma MJ (2008), Inapparent infections and cholera dynamics. *Nature* 454, 877–880.

[19] Warrell DA (2002), Clinical features of malaria. In *Essential Malariology* (Warrell DA and Gilles HM eds), pp. 191–205, Oxford University Press.
