# Supplementary figures and images for: The Potential Elimination of Plasmodium vivax Malaria by Relapse Treatment: Insights from a Transmission Model and Surveillance Data from NW India
Source: PLoS Negl Trop Dis. 2013 Jan 10;7(1):e1979. doi: 10.1371/journal.pntd.0001979 (PMC3542148; doi:10.1371/journal.pntd.0001979)

Fig.S1

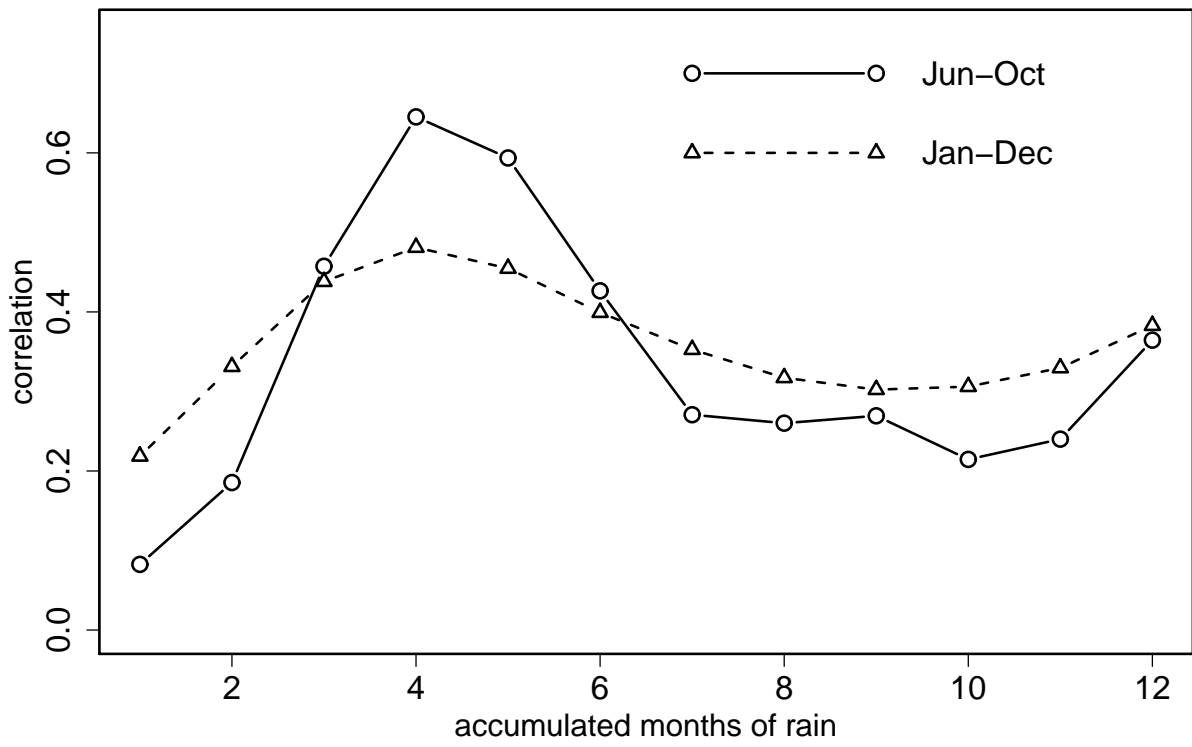

Supplement: Figure S1 — Correlation between cases and accumulated rain. The correlation is significantly higher in the months between June and October (solid line + circle) during the monsoon season, than when all twelve months are considered (dashed line + triangle), due to the confounding effects of relapse cases in the latter. (PDF) [file pntd.0001979.s002.pdf]

Fig.S2

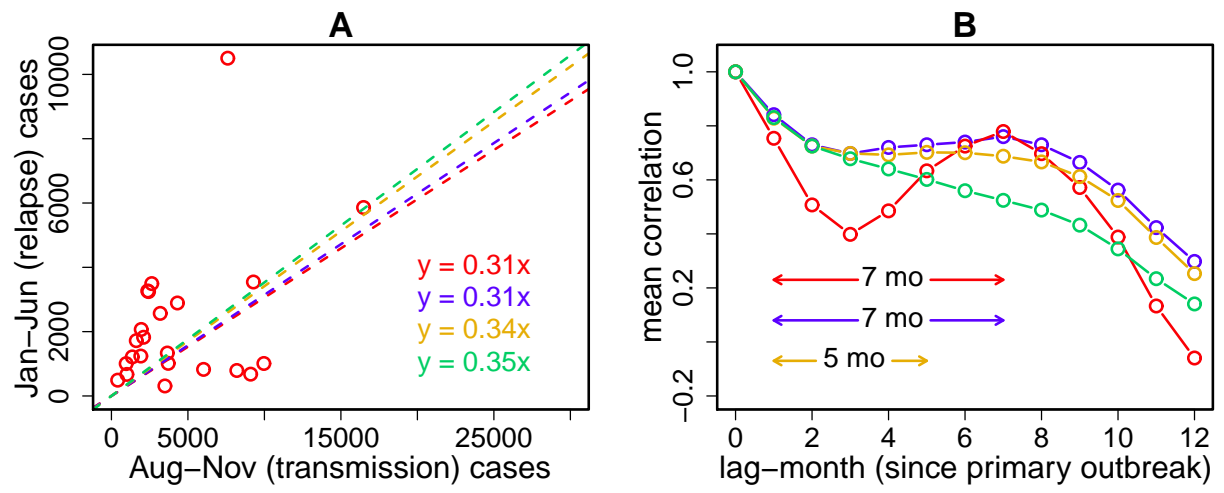

Supplement: Figure S2 — Comparison between data (red) and models SEIH3QS (blue), SEIQS (yellow) and SEIRS (green). Each model is simulated 1000 times. A. A linear regression of aggregated transmission and relapse cases gives relapse rates of 34% and 35% for the two non-relapse models SEIQS and SEIRS, respectively, both values higher than the 31% rate estimated from the SEIH3QS model and suggested by the data. Data points (red open circles) are also shown. B. The SEIQS model has a shorter mean latency of 5 months, (and the SEIRS model by construction has none), compared to the 7-mo latency estimated by the SEIH3QS model and consistent with the data. (PDF) [file pntd.0001979.s003.pdf]

Fig.S3

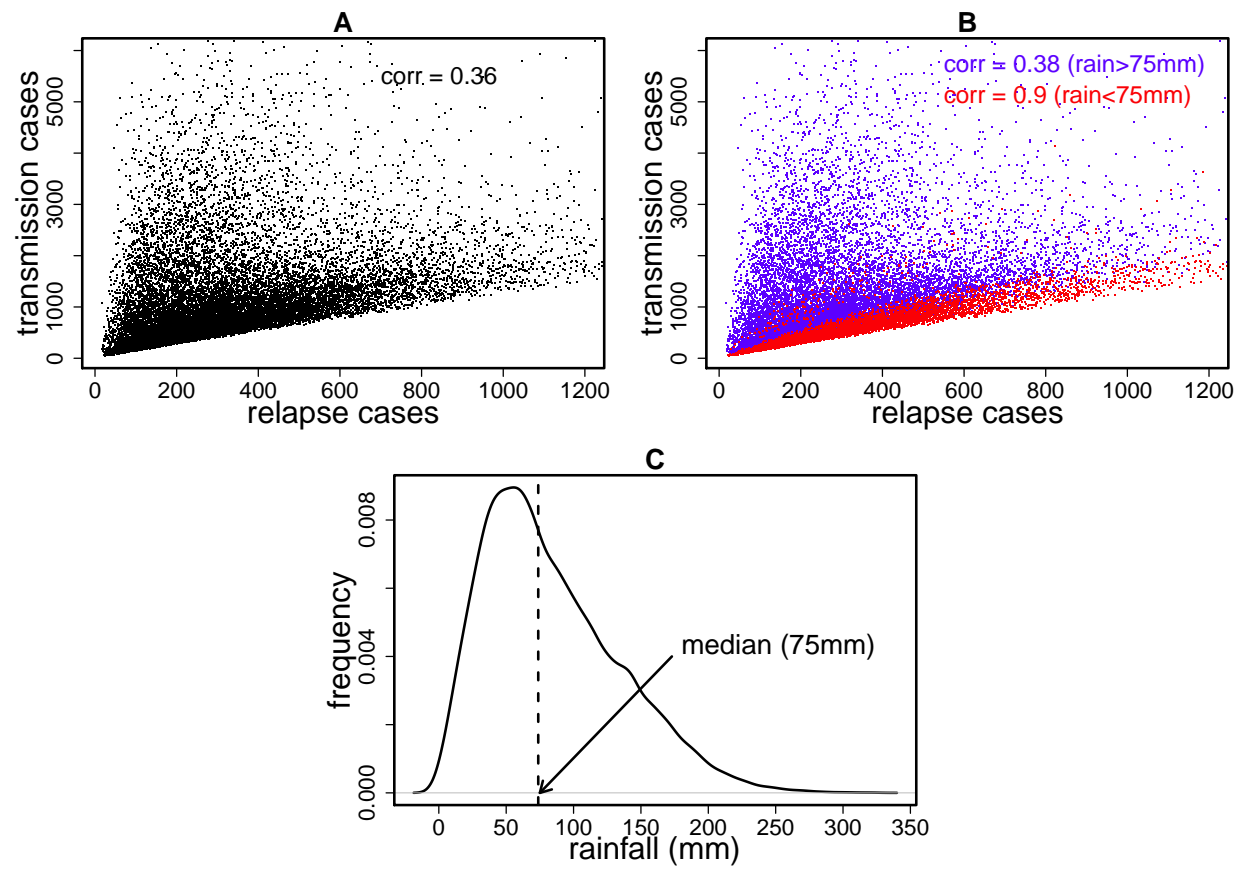

Supplement: Figure S3 — Correlation of relapse and transmission cases. The SEIH3QS model is simulated (using the MLE parameters) 25 yrs ahead with 1000 surrogate rainfall series, and Jan–Jun relapse cases, and the Aug–Nov transmission cases are aggregated for each simulated year, giving 25,000 paired values. A. A scatterplot of these (aggregated) relapse and transmission case pairs does not exhibit a meaningful correlation because of the confounding effects of rainfall. B. Partitioning by low (red) and high (blue) rainfall years, defined by total monsoon rain (aggregated between June–Sept) < and >75 mm, reveals a strong positive correlation during low-rainfall years. C. The (aggregated) rainfall distribution has a median of 75 mm, giving the same number of red and blue points in figure B. (PDF) [file pntd.0001979.s004.pdf]

Fig.S4

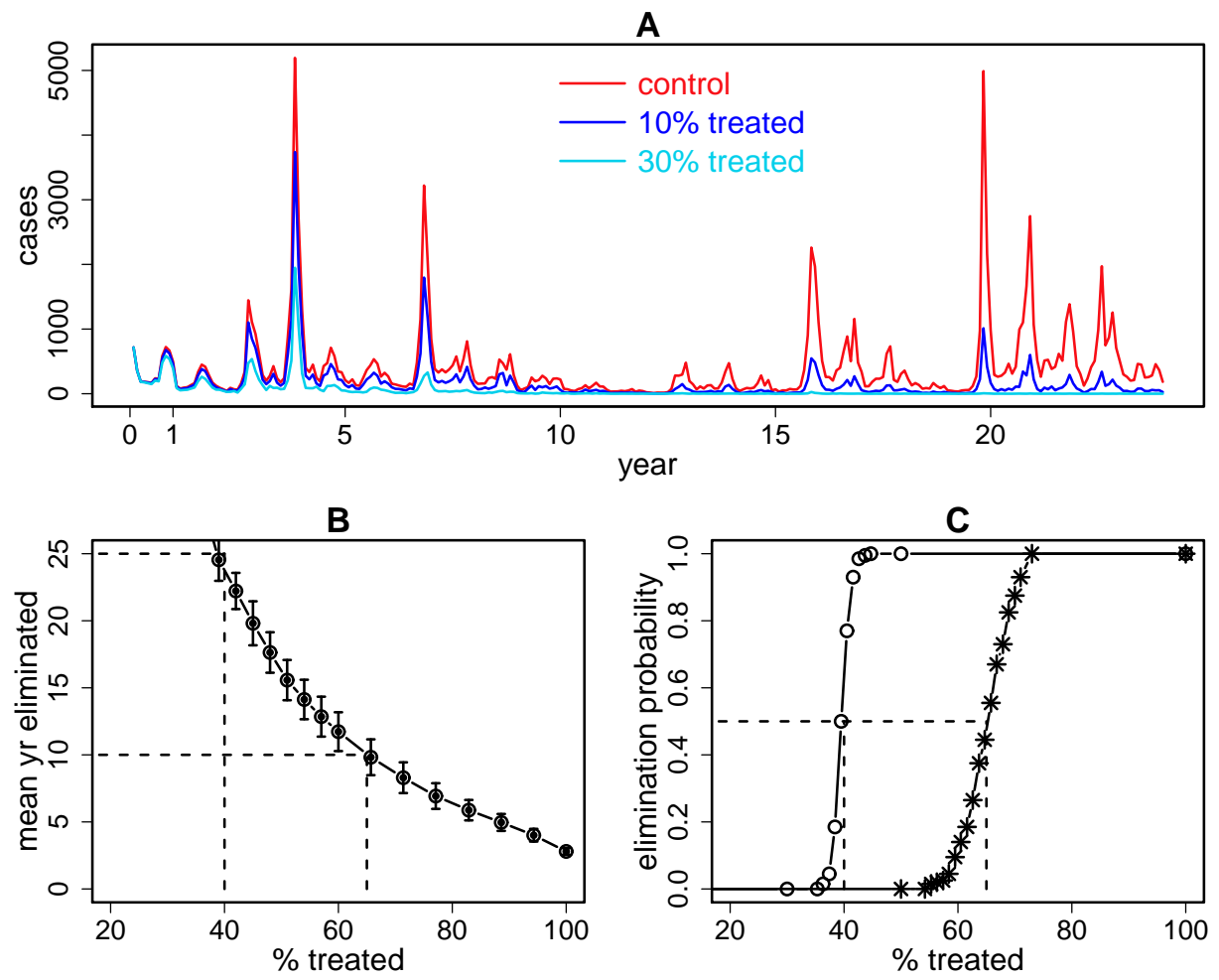

Supplement: Figure S4 — Relapse treatment and elimination probability. Elimination is computed as the year-long absence of P. vivax malaria cases for every year starting in a given year and lasting for the entire simulation length ( = 1000 yrs). A. Representative examples of simulated Pv time series for the three treatment levels used in Fig. 3A, a = 0, 0.1, 0.3, using a single surrogate rainfall. Progressive suppression of cases at 30% treatment level (light blue) suggests the possibility of P. vivax elimination in near future. B. Plot shows mean year of P. vivax elimination as a function of treatment level (a), indicating that 40% treatment can eliminate the parasite in 25 years, and 65% treatment can achieve the same within a decade (mean and error bars are computed over 200 surrogate rainfall series). C. Plot shows the probability of elimination in 25 yrs (circles) and 10 yrs (stars), versus treatment level, corroborating that 40% and 65% relapse treatment can eliminate P. vivax malaria in 25 yrs and 10 yrs respectively (at probability P = 0.5). (PDF) [file pntd.0001979.s005.pdf]

Fig.S5

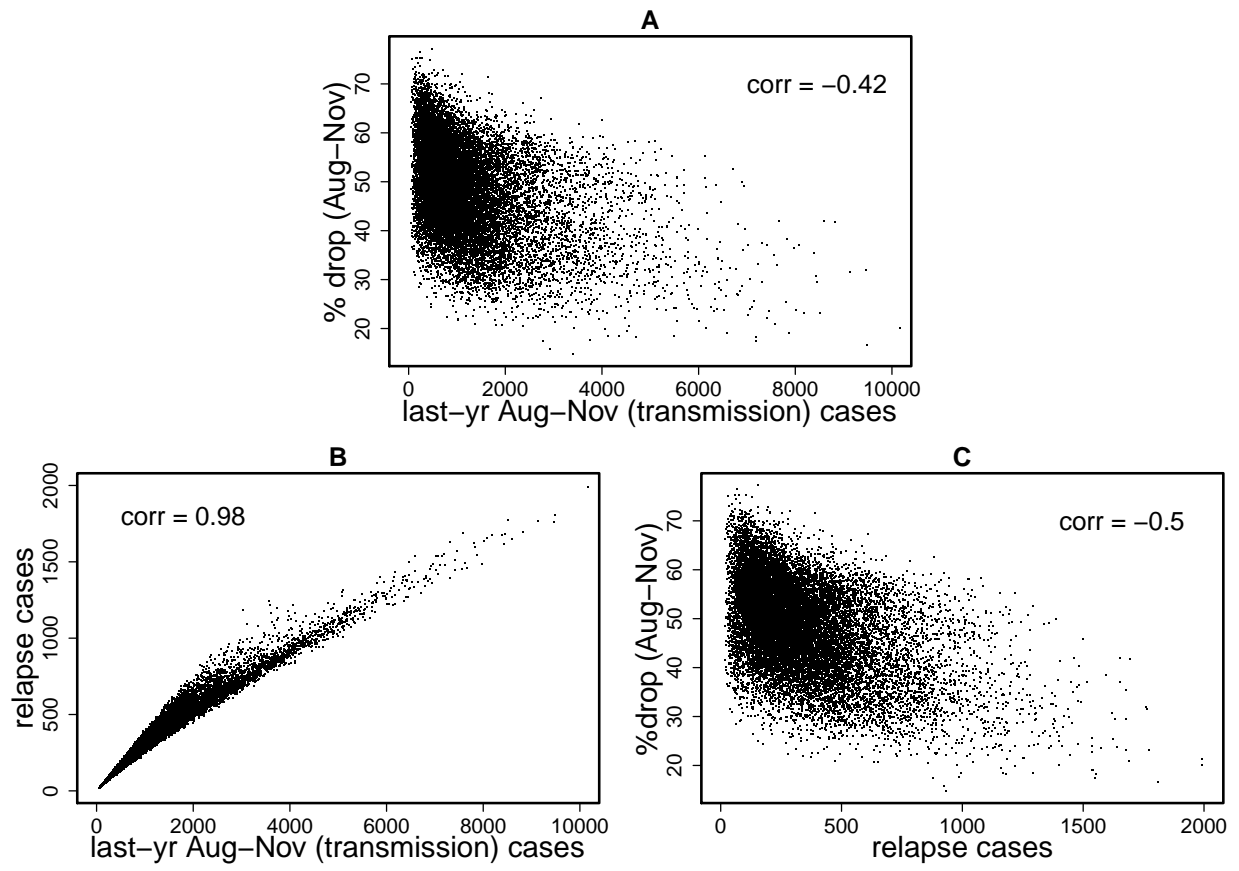

Supplement: Figure S5 — Correlation of treatment effect and preceding transmission intensity. The treatment model S1 is simulated (using MLE parameters) at a 10% treatment level (a = 0.1), 25 yrs ahead with 1000 surrogate rainfall series. A. A scatterplot of % drop in current transmission intensity vs preceding (aggregated) transmission cases shows a negative correlation. This inverse relationship arises from a strong positive correlation between these earlier transmission cases and current relapse cases (B), which in turn correlate negatively with the % drop (C), presumably because of the parasite's dependence on relapse to survive during the low-transmission season. (PDF) [file pntd.0001979.s006.pdf]
