# Supplementary material for: The Potential Elimination of Plasmodium vivax Malaria by Relapse Treatment: Insights from a Transmission Model and Surveillance Data from NW India
Source: PLoS Negl Trop Dis. 2013 Jan 10;7(1):e1979. doi: 10.1371/journal.pntd.0001979 (PMC3542148; doi:10.1371/journal.pntd.0001979)
Supplement: Table S1 — Parameter definition and maximum likelihood estimates (MLEs) for the different models, SEIH3QS, SEIH6QS and SEIQS. Parameters not estimated are denoted with superscript *. Setting μ EI = 24/yr assumes an average 15-day human incubation period before clinical symptoms arise [11]. (PDF) [file pntd.0001979.s007.pdf]

Table S1

Symbol	Definition	Unit	SEIH^{3}QS	SEIH^{6}QS	SEIQS
$\mu_{EI}^{(*)}$	E→I transition rate	yr^{-1}	24	24	24
μ_{IH}	I→H transition rate	yr^{-1}	11.958	24.662	–
μ_{HI}	H→I transition rate	yr^{-1}	1.690	1.727	–
μ_{IS}	I→S transition rate	yr^{-1}	50.000	3.814	2.138
μ_{IQ}	I→Q transition rate	yr^{-1}	11.527	129.332	119.367
μ_{QS}	Q→S transition rate	yr^{-1}	67.511	34.445	2.879
n	number of H classes	–	3	6	0
q	relative infectivity of Q class	–	0.001	0.001	0.009
τ	mean lag for mosquitoes	day	7.38	7.056	5.472
ρ	case reporting fraction	–	0.033	0.0311	0.055
σ_{pro}	s.d. of dynamic noise	yr$^{1/2}$	0.124	0.110	0.077
σ_{obs}	s.d. of measurement noise	–	0.205	0.195	0.215
b_r	coef of rainfall covariate	–	0.253	0.210	0.214
$S(0)$	initial fraction in S class	–	0.379	0.368	0.991
$E(0)$	initial fraction in E class	–	0.001	0.001	0.0003
$I(0)$	initial fraction in I class	–	0.011	0.002	0.003
$H_1(0)$	initial fraction in H_1 class	–	0.001	0.001	–
$H_2(0)$	initial fraction in H_2 class	–	0.001	0.0002	–
$H_3(0)$	initial fraction in H_3 class	–	0.001	0.001	–
$H_4(0)$	initial fraction in H_4 class	–	–	0.0003	–
$H_5(0)$	initial fraction in H_5 class	–	–	0.001	–
$H_6(0)$	initial fraction in H_6 class	–	–	0.002	–
$Q(0)$	initial fraction in Q class	–	0.606	0.624	0.006
$\kappa_1(0)$	initial $\kappa_1(t)$	–	0.156	0.004	0.022
$\kappa_2(0)$	initial $\kappa_2(t)(\equiv \mu_{SE}(0))$	–	0.045	0.0001	0.014
b_1	1st spline coefficient	–	2.458	3.409	3.430
b_2	2nd spline coefficient	–	4.468	5.107	4.672
b_3	3rd spline coefficient	–	3.987	4.715	4.482
b_4	4th spline coefficient	–	4.862	5.506	4.986
b_5	5th spline coefficient	–	3.695	4.611	4.239
b_6	6th spline coefficient	–	4.653	5.374	4.840
$1/\delta^{(*)}$	mean human life span	yr	50	50	50
a	fraction treated for relapse	–	variable	–	–
