# Supplementary material for: The Potential Elimination of Plasmodium vivax Malaria by Relapse Treatment: Insights from a Transmission Model and Surveillance Data from NW India
Source: PLoS Negl Trop Dis. 2013 Jan 10;7(1):e1979. doi: 10.1371/journal.pntd.0001979 (PMC3542148; doi:10.1371/journal.pntd.0001979)
Supplement: Table S2 — MLEs for the SEIH3QS parameters and their lower and upper bounds of approximate 95% confidence intervals (see Table S1 for units; pre-set parameters are denoted with superscript *. CI values of 0, 1 and ∞ correspond to confidence intervals reaching the boundaries of parameter space. (PDF) [file pntd.0001979.s008.pdf]

Table S2

Parameter	MLE values	95% CI
$\mu_{EI}^{(*)}$	24	(—,—)
μ_{IH}	11.958	(3.97,58.86)
μ_{HI}	1.690	(1.437,2.073)
μ_{IS}	50.000	(0,∞)
μ_{IQ}	11.527	(0,∞)
μ_{QS}	67.511	(0,∞)
q	0.001	(0,1)
τ	7.38	(3.6,14.04)
ρ	0.033	(0.01,1)
σ_{pro}	0.124	(0.096,0.179)
σ_{obs}	0.205	(0.175,0.254)
b_r	0.253	(0.148,0.395)
$S(0)$	0.379	(0.01,0.834)
$E(0)$	0.001	(0.0002,0.007)
$I(0)$	0.011	(0.003,0.093)
$H_1(0)$	0.001	(0,0.011)
$H_2(0)$	0.001	(0,0.009)
$H_3(0)$	0.001	(0,0.008)
$Q(0)$	0.606	(0.345,0.89)
$\kappa_1(0)$	0.156	(0,∞)
$\kappa_2(0)$	0.045	(0,∞)
b_1	2.458	(1.415,4.435)
b_2	4.468	(4.015,5.773)
b_3	3.987	(2.791,5.218)
b_4	4.862	(3.815,6.112)
b_5	3.695	(2.346,5.022)
b_6	4.653	(3.875,5.884)
$1/\delta^{(*)}$	5	(—,—)
